# Metabolism as means for hypoxia adaptation: metabolic profiling and flux balance analysis

**DOI:** 10.1186/1752-0509-3-91

**Published:** 2009-09-09

**Authors:** Jacob D Feala, Laurence Coquin, Dan Zhou, Gabriel G Haddad, Giovanni Paternostro, Andrew D McCulloch

**Affiliations:** 1 Burnham Institute for Medical Research, 10901 North Torrey Pines Road, La Jolla, CA 92037, USA; 2 Department of Pediatrics, University of California, San Diego, 9500 Gilman Drive 0735, La Jolla, CA 92093, USA; 3 Department of Bioengineering, University of California, San Diego, 9500 Gilman Drive, 0412, La Jolla, CA 92093-0412, USA

## Abstract

**Background:**

Cellular hypoxia is a component of many diseases, but mechanisms of global hypoxic adaptation and resistance are not completely understood. Previously, a population of *Drosophila *flies was experimentally selected over several generations to survive a chronically hypoxic environment. NMR-based metabolomics, combined with flux-balance simulations of genome-scale metabolic networks, can generate specific hypotheses for global reaction fluxes within the cell. We applied these techniques to compare metabolic activity during acute hypoxia in muscle tissue of adapted versus "naïve" control flies.

**Results:**

Metabolic profiles were gathered for adapted and control flies after exposure to acute hypoxia using ^1^H NMR spectroscopy. Principal Component Analysis suggested that the adapted flies are tuned to survive a specific oxygen level. Adapted flies better tolerate acute hypoxic stress, and we explored the mechanisms of this tolerance using a flux-balance model of central metabolism. In the model, adapted flies produced more ATP per glucose and created fewer protons than control flies, had lower pyruvate carboxylase flux, and had greater usage of Complex I over Complex II.

**Conclusion:**

We suggest a network-level hypothesis of metabolic regulation in hypoxia-adapted flies, in which lower baseline rates of biosynthesis in adapted flies draws less anaplerotic flux, resulting in lower rates of glycolysis, less acidosis, and more efficient use of substrate during acute hypoxic stress. In addition we suggest new specific hypothesis, which were found to be consistent with existing data.

## Background

Hypoxia is a component of many diseases and can have devastating effects on the cell. A major goal of medical research is to design therapies to enhance cellular defenses to hypoxic insult, but more basic research on the mechanisms of hypoxic cell death and defense systems is needed as a prerequisite.

Metabolically, the primary effect of reduced oxygen levels is a dramatic drop in respiratory activity in the mitochondria. The cell is forced to depend on glycolysis for ATP, though glycolytic ATP production is only a fraction of mitochondrial output, and acidosis occurs as mitochondrial consumption of protons slows [1]. The loss of ATP and increased acidosis may cause damage by a number of mechanisms and imbalances [2-5], but it is not conclusively known which of these is responsible for cell death.

Systems biology seeks to build quantitative computer models of biological networks, in order to derive testable hypotheses from large and complex datasets [6]. Recently, genome sequencing and other high-throughput technology has allowed researchers to integrate multiple genome-wide datasets to build global models of cellular activity [7]. The cellular response and defenses to hypoxia are far-reaching and complex [8], therefore our understanding of these mechanisms may also benefit from a global systems approach.

*Drosophila melanogaster *has been a common model organism both for systems biology approaches and for hypoxia research because of the well-known genome, useful tools for genetic manipulation, fecundity and short lifespan of the fruitfly, as well as for its innate tolerance to extreme fluctuations in oxygen levels [9]. In addition, many hypoxia response genes are shared between flies and humans [10,11]. We previously built a network reconstruction of central metabolism using the fly genome, and then integrated microarray and metabolomic data to refine the network to represent reactions active in hypoxic muscle tissue. In separate studies we then simulated fluxes within the network in order to study mechanisms of hypoxia tolerance in normal [12] and aging [13] flies.

Here we apply a similar technique to a population of flies that we had previously adapted for enhanced hypoxia tolerance using experimental selection [14]. We hypothesize that this special population of hypoxia-adapted flies regulate central metabolism in novel ways, in addition to other mechanisms for hypoxia tolerance discovered in past studies [14,15]. However, metabolic adaptations to hypoxia in adapted flies are completely unknown, and because of the random nature of directed evolution, changes that confer improved function can be completely unexpected. Therefore, new observations are needed to help generate new hypotheses for possible mechanisms, and these measurements should be made from a global perspective. We propose a discovery-based strategy to find global flux differences in this population versus a control, in order to find targets for more detailed follow-up using classical biochemical and molecular biology methods. Basic science understanding of novel mechanisms for hypoxia tolerance in this special population of flies may spur future translational applications, but this is not directly a disease model.

In this study we applied ^1^H NMR spectroscopy to measure metabolic profiles for adapted and naïve (control) flies, then examined these profiles by multivariate analysis to find the main sources of metabolite variation and observe how the groups clustered and shifted within multidimensional space under hypoxic stress.

Next, we approximated fluxes by the difference between hypoxia and baseline measurements, fitted the model to these fluxes as constraints, and compared ATP efficiency and proton accumulation between adapted and naïve flies. Enzyme-controlled fluxes were compared across groups, and similarly to our previous work we found differences in fluxes of pyruvate metabolism, though the specific mechanisms were different. Finally, we compared fluxes in the model to differential expression of genes in the microarray data, which pointed to enzymes involved in pyruvate metabolism. Here we have only examined aspects of adaptation present in central ATP-producing metabolism. Other studies have examined non-metabolic aspects [14,15] and research is ongoing in these areas.

## Results

### Experimental overview

**Figure 1:**
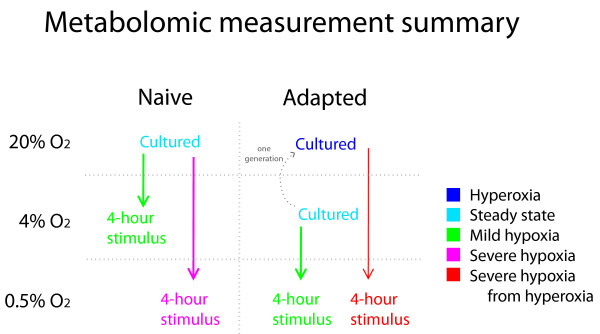
**Summary of experimental conditions**. Summary of the experimental conditions for the 7 metabolic profiles. Measurements were gathered for both males and females in all conditions illustrated, for a total of 14.

We gathered metabolic profiles for hypoxia-adapted and naïve control flies under their respective chronic culture conditions (4% and 20% oxygen, respectively) and under acute hypoxia (4 hours at 0.5% oxygen). Next, acute hypoxia stimuli were applied, with the assumption that a 4-hour timescale would long enough for metabolism to adapt to a new steady state, but short enough to avoid gene expression changes. We measured naïve flies at 4% (after 4 hours) to compare with adapted flies in their normal state. Then, adapted flies were measured after culturing for one generation in room air and then subjected to acute hypoxia (4 hours at 0.5%), in order to compare acute stress over a similar step size (from 20% to 0.5% O_2_). For simplicity and to differentiate the hypoxic stress conditions, we labeled the experimental conditions as "steady state" for chronic culture conditions, "perceived hyperoxia" for adapted flies cultured in room air, "mild hypoxia" for smaller steps in oxygen (moving adapted flies from 4% to 0.5% and naïve flies from 20% to 4% O_2_), "severe hypoxia" for the large step size (naïve flies from 20% to 0.5% O_2_), and "severe hypoxia from hyperoxia" for the adapted flies cultured in room air and subjected to hypoxia. Males and females were measured separately for each experiment. The experimental groups are summarized in Figure 1.

In 0.5% oxygen, the condition for which simulations were run, both populations were in a stupor and not able to fly. However, recovery of function from anoxia is indeed faster in the adapted population [14], and adapted flies were observed to have more activity in 4% oxygen than controls, though physical activities such as flight have not been quantified.

### Raw concentration profiles

Thirty-five metabolites appeared in 14 different metabolic profiles (males and females from the 7 experiments listed in Figure 1), making the data difficult to examine by direct inspection. One trend, however, is readily apparent in the raw data. Except for the "severe hypoxia from hyperoxia" group, which had different behavior than all other groups as explained in the next section, hypoxic stress tended to increase the levels of most metabolites including free glucose. This is consistent with previous experiments and implies that, since not every metabolite can be an anaerobic end product, substrates such as starches and proteins are broken down into monomers for fuel faster than they can be consumed by catabolic pathways. Raw concentration profiles for the greatest changing metabolites are provided [see Additional file 1, Figure S1].

### Principal component analysis

Principal component analysis (PCA) is a mathematical technique that can reduce the dimensionality of the data and provide an unbiased determination of which metabolites vary the most across groups, and by how much. When the 14 metabolic profiles were decomposed by PCA, the first three Principal Components (PCs) accounted for 90% of the variation in the data [see Additional file 1, Figure S2]. Each PC is composed of several weighted metabolite contributions. Table 1 lists the top three PCs and the compounds contributing more than 5% weight. These are the "basis sets" of metabolites that cause most of the variation between samples. Each experimental measurement can be decomposed into a baseline metabolite profile plus some linear combination of these principal components specific to that measurement.

**Table 1:** Principal component weightings for the metabolomic data

**PC 1: 61%**		**PC 2: 18%**		**PC 3: 11%**	
***Metabolite***	***Weight***	***Metabolite***	***Weight***	***Metabolite***	***Weight***
Glucose	0.491	alanine	0.458	oxalacetate	0.187
alanine	0.456	glucose	0.370	glucose	0.183
oxalacetate	0.389	lactate	-0.128	alanine	0.127
glutamate	0.325	oxalacetate	-0.783	acetate	-0.952
b-alanine	0.298				
acetate	0.228				
lactate	0.225				

Metabolite weighting in top three Principal Components (contributing at least 5% of PC total).

The first Principal Component had a wide, flat distribution of weights for almost all metabolites, meaning that most of the variation during hypoxia was due to a global increase in free compound levels, which is the expected result as the cells break down macromolecules such as starches and proteins in order to free stored energy (this trend is apparent in the raw data). Principal components 2 and 3 account for variation in key metabolites such as oxalacetate, glucose, lactate, alanine, and acetate, which are produced in varying amounts in the different groups.

**Figure 2:**
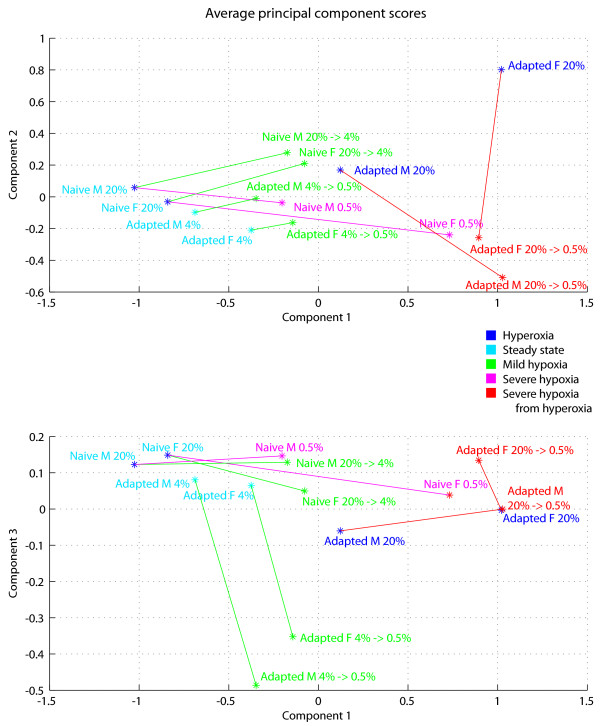
**Principal component analysis of the metabolic profiles**. Average group scores along the first three principal components (PCs). Lines represent shifts from 4-hour hypoxia stimulus. Groups are separated by color according to the code in Figure 1. Figure 2 plots average scores for all 14 groups within the first 3 PC dimensions. The top axes display the average scores along PC 1 and PC 2, and the bottom axes plot PC 1 versus PC 3. Lines are drawn to connect steady state profiles for each group with profiles under acute hypoxia conditions. The direction and magnitude of these lines can be directly mapped to changes in specific metabolites using Figure S1 in the supplementary data.

Furthermore, the experimental groups cluster in patterns in the Principal Component space that can provide interesting biological insight. This technique can be used to visualize the different experimental groups and their hypoxic responses in multidimensional space, as shown in Figure 2, and Table 1 can be used to trace movements within this space back to specific metabolites. Figure 2 shows that "steady state" profiles - groups measured in their chronic level of O_2 _- tend to cluster in the same region in PC space. In contrast, the "perceived hyperoxia" group (adapted flies cultured at 20% O_2_) appeared in the same region of the metabolic space as the groups measured under hypoxic stress. In other words, adapted flies in 4% oxygen have similar metabolic signatures as normal flies in normoxia, but when adapted flies are cultured in normoxia they resemble a stressed state. This result hints that the metabolic adaptations of the adapted flies are specifically tailored to their chronic level of oxygen, rather than to oxygen fluctuations in general (presumably a stable genetic modification since this group was cultured from embryo in normoxia),

Because the "perceived hyperoxia" group (adapted flies in normoxia) appeared to be in a stressed state, had more variable NMR metabolomic data both before and after hypoxia, and differed widely for males versus females, this group is excluded in the presentation of flux-balance analysis in the next section. However, data for all groups, including "severe hypoxia from hyperoxia," are provided [see Additional file 2].

For all other experiments, males and females in the same group tended to cluster together in PC space and their hypoxic shifts were often similar. The overall similarity of metabolic signatures between males and females lends some confidence to the precision of the NMR data for these groups. Therefore, for simplicity, presentation of flux-balance analysis in the next section will be limited to males, though the general conclusions were found to hold for both sexes. Data for both sexes are made available [see Additional file 2].

For the three experiments subjecting "steady state" groups to 4-hour hypoxia (naïve flies in 4% and 0.5% oxygen and adapted flies in 0.5% oxygen), we used flux-balance analysis to examine network-level features of metabolic changes.

### Overview of simulation conditions

Important metabolites for energy metabolism such as NADH, ATP, ADP, acetyl-CoA, and creatine were not seen in sufficient concentrations in the ^1^H NMR spectra to quantify, however, they are all present as important variables within the model. The model links mass substrate fluxes to all of these metabolites via a stoichiometrically precise reaction network that includes and therefore balances NAD+ and NADH. Also important is the timescale of the experiments. Creatine and phosphocreatine (and more importantly the analogous arginine and phosphoarginine system in flies [16]) are important as a buffer for short-term fluctuations in ATP/ADP ratios, and adenylate kinase similarly protects ATP/AMP ratios in the short term. However, we have chosen a 4-hour timescale to emphasize steady-state fluxes over short-term feedback, and so even though these reactions exist in the model, they are not used.

Ten metabolites were chosen for fitting the model, based on the requirements that they (1) were present in the NMR spectra in sufficient concentrations for all experimental conditions, (2) were represented in our metabolic reconstruction, and (3) changed concentration (accumulated or dissipated) in a direction that was feasible with the model. Fluxes were approximated by dividing differences in two concentrations by the experiment time (4 hours), and standard errors for fluxes were derived by adding the variances of the two concentration measurements.

Glycogen availability was left unconstrained for all simulations. This assumption was supported by the large increase in free glucose for most hypoxic measurements, indicating that glycogen and trehalose breakdown supplied glucose monomers faster than the system could use them. Reactions for fatty acid catabolism are present in the model, but literature data has suggested that flight muscle metabolism in closely related insects (order Diptera) is almost completely based on carbohydrates [17,18]. The large deposits of glycogen in flight muscle of flies, the depletion of these reserves after prolonged flights, and the rapid catabolism by flight muscle *in vitro*, indicate that glycogen is the carbohydrate that provides the major source of energy for meeting the metabolic requirements of active flight [19]. In hypoxia, we have seen large depletion of the glycogen reserves as well, suggesting this remains the major source of energy in the face of oxygen fluctuations [13]. However, since lipids are difficult to distinguish and quantify using ^1^H NMR, we have not measured whether fats or lipids remain unused as an energy source during hypoxia. Lipids could be measured in future experiments, for example using gas chromatography followed by mass spectrometry (GC-MS), in order to confirm this.

We did not have measurements of oxygen consumption, which presented a modeling challenge. Since our flux-balance model optimizes for ATP production, the combination of unconstrained glycogen and unconstrained oxygen would simply produce infinite fluxes of both. Instead, for each experiment we swept the oxygen consumption constraint over ranges that encompassed the qualitative features of the model.

### Oxygen consumption requirements in simulation

Every simulation required some minimum level of oxygen to produce the organic end products observed in the NMR spectra, below which the model was infeasible. The minimum feasible oxygen uptake was less than 5 nmol O_2 _per minute per mg protein for all three simulations, much lower than physiological measurements of normoxic oxygen uptake on the order of 1,000 and 3,000 nmol O_2 _per minute per mg protein in mitochondria, and per mg dry weight in whole flies, respectively [20,21]. In hypoxia at 4% O_2_, oxygen consumption was previously measured roughly on the order of 2,200 nmol O_2 _per mg [14], deviating only a small amount between naïve and adapted flies, and still two orders of magnitude higher than the minimum uptake suggested by the model. A trend of lower minimum oxygen consumption in adapted flies can be seen [see Additional file 1, Figure S3], possibly suggesting greater flexibility in regulating oxygen demand, but this trend was not statistically significant for the three groups tested.

### Key hypoxia tolerance indicators

At the physiological ranges of oxygen uptake noted above, mitochondrial respiration still dominates central metabolism, masking subtler differences in fluxes. Therefore, to focus on interesting differences between groups and since we do not have measurements of true oxygen uptake "operating points" for each group, we compared ATP production across models using a common oxygen uptake that was as low as possible while still producing a feasible result for all simulations (O_2 _uptake = 4.8 nmol/min/mg protein). The true flux distributions in the organism are a superimposition of the hypoxic pathways shown, plus some large flux through glycolysis and mitochondrial respiration. Since respiration is neutral in terms of protons and produces no end products besides CO_2_, and also since small changes in ATP production rates can have major effects in concentration over the long term, hypoxic flux patterns shown here are likely to be important for hypoxia tolerance even though their magnitudes are small relative to the concurrent high levels of respiration seen in physiological conditions.

**Figure 3:**
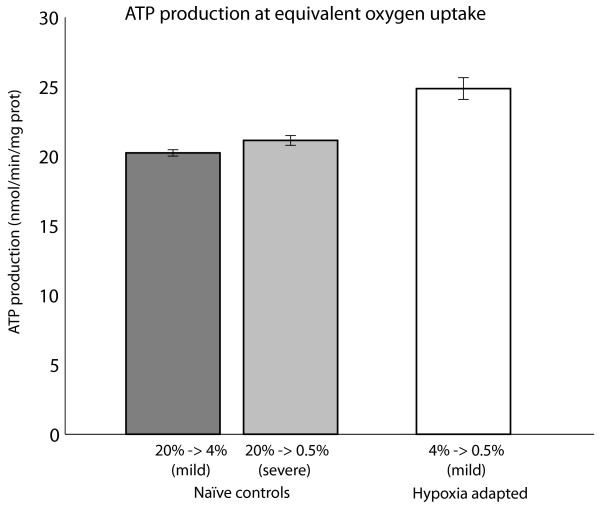
**ATP production in simulations**. Simulations of ATP production at a common O_2 _uptake rate (4.8 nmol/min/mg protein) for groups starting from their steady state concentrations (20% for naïve controls and 4% for adapted flies). For equivalent oxygen uptake, adapted flies are able to produce more ATP than naïve controls (p < 0.05 for all comparisons of the three groups). The O_2 _uptake rate was set near the minimum allowed uptake for all three simulations to produce feasible results. Error bars indicate standard deviation of 100 simulations incorporating the variances of NMR measurements, as detailed in the text. For simplicity, data are shown for males only.

In adapted flies ATP production is higher at a common O_2 _uptake than both groups of naïve flies (p < 0.05, see Figure 3), and experiments sweeping the oxygen constraint suggest that this holds for any given O_2 _uptake rate [see Additional file 1, Figure S3]. As a corollary, O_2 _consumption is also lower in adapted flies for any given ATP output. Therefore, although we did not have measurements of oxygen consumption for each group, simulations suggest that the hypoxia-adapted metabolism is more efficient in terms of ATP/O_2_, regardless of where the O_2 _"operating point" may lie.

**Figure 4:**
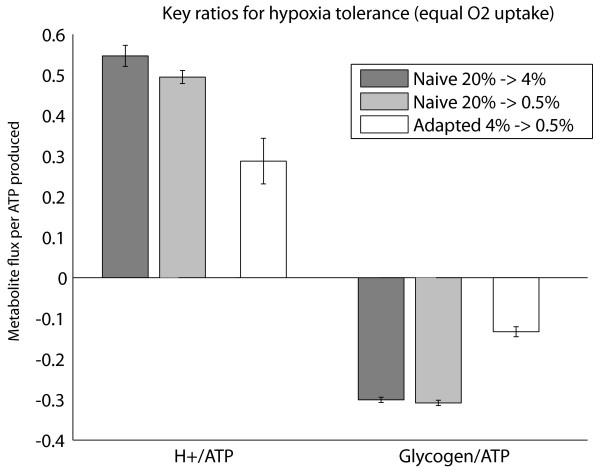
**Key measures of hypoxia tolerance in simulations**. Production of key ratios for hypoxia tolerance at a common O_2 _uptake rate (4.8 nmol/min/mg protein). Naïve flies have lower proton/ATP and substrate/ATP ratios than adapted flies at any given oxygen supply (p < 0.05 for all comparisons of adapted versus the two naïve control groups, and p < 0.05 for differences in H+/ATP ratio between the two controls). Error bars indicate standard deviation of 100 simulations incorporating the variances of NMR measurements. H+ stands for proton accumulation. For simplicity, data are shown for males only.

Key ratios of hypoxia tolerance were compared across groups at this common oxygen uptake rate. As shown in Figure 4, proton production per ATP (H+/ATP) was lower in adapted flies as well as substrate efficiency - adapted flies consumed less glycogen substrate per ATP (Glycogen/ATP) than naïve flies. These results hold for females, and when not normalized for ATP production. The differences are more pronounced at lower oxygen uptake, but hold true for all O_2 _uptake rates.

Proton production within the model is complex. A recent review describes well the detailed mechanism for proton production by ATP hydrolysis during hypoxia [22]. In it the authors use the stoichiometry of proton handling to argue that it is the hydrolysis of the two ATP created by glycolysis that produces a proton each, and the pyruvate to lactate reaction actually consumes one proton, resulting in a net +1 proton for conversion of glucose to lactate. On the other hand, each pyruvate that is diverted through PDH for mitochondrial oxidation consumes two net protons, which balances the protons created by hydrolysis of ATP produced in forming that pyruvate molecule. Therefore, each pyruvate diverted out of the mitochondrion for conversion to lactate (or to any end product, e.g. alanine) decouples this zero proton balance and accumulates acidosis in the cytoplasm [22]. Our model accounts for the proton stoichiometries of all of the key transporters and reactions involved in ATP production and ATP consumption, and shows the behaviour just described. From this perspective, and within our model, it is the degree of decoupling of glycolysis from mitochondrial respiration that produces acidosis, rather than the creation of lactate as the specific end product.

### Comparison of active pathways

**Figure 5:**
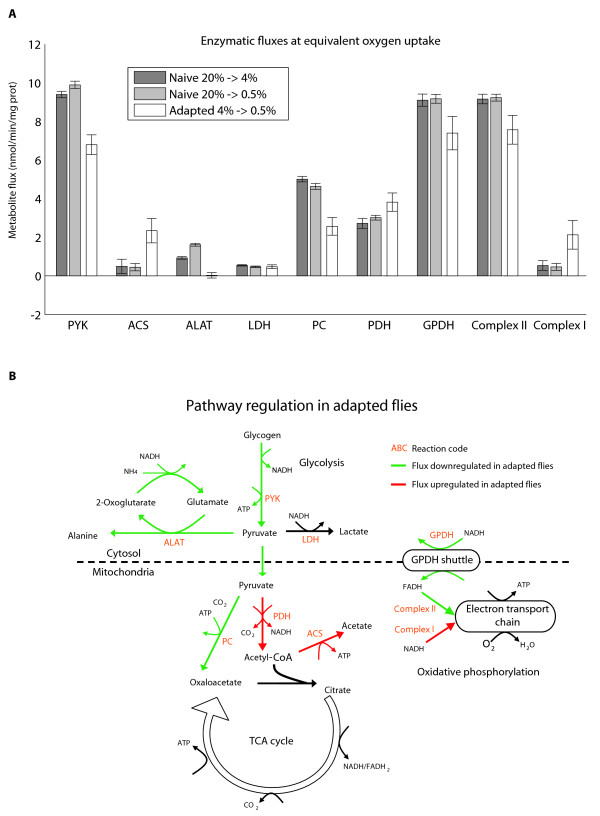
**Pathway differences between adapted and naïveflies**. **A) **Fluxes through key reactions at a common oxygen uptake. Compared with naïve flies, simulations of adapted flies showed lower glycolysis, pyruvate carboxylase, GPDH, and Complex II activity, and higher fluxes through Complex I. All tests between the adapted group and the two naïve groups had p < 0.05, except for LDH, which is shown for comparison only. Error bars indicate standard deviation of 100 simulations incorporating the variances of NMR measurements. **B) **Cartoon of differences in fluxes in simulations of adapted flies versus naïve controls. In naïve flies, oxalacetate production in the NMR profiles drives increased activity of glycolysis, pyruvate carboxylase, and Complex II of the electron transport chain. *Abbreviations*: PYK - pyruvate kinase, ACS - acetyl-CoA synthase, ALAT - alanine transaminase (cytosolic), LDH - lactate dehydrogenase, PC - pyruvate carboxylase, PDH - pyruvate dehydrogenase, CS - citrate synthase, SuccDH - succinyl CoA dehydrogenase (TCA cycle), MDH - malate dehydrogenase (mitochondrial), GPDH - glycerol-3-phosphate dehydrogenase shuttle, Complex II - succinyl CoA dehydrogenase (electron transport chain), Complex I - NADH dehydrogenase.

We inspected differences in enzyme fluxes at this simulated oxygen uptake. Each experimental group likely operated at a different O_2 _uptake, but for the reasons argued above, simulations were again compared at minimum feasible O_2 _for all groups. As with the previous two figures, Figure 5A depicts flux simulations generated from NMR data in males for the three experimental groups that started at steady state culture conditions. All reactions with nonzero fluxes that were not transport or exchange reactions (i.e. enzyme-linked transformation reactions) were ranked for the largest differences across experimental groups. Fluxes with the largest difference across these groups are shown.

The values presented in Figure 5A are fluxes rather than concentrations. Hypoxic production of metabolites in insect flight muscle is not well documented in the literature for comparison, although whole alder leaf beetles produced just over 4 μmol/g lactate after 10 hours, which is approximately 6 pmol/min/mg [22]. These were calculated from wet weight rather than dry weight, which might partially account for the discrepancy with our measurements. Also, when compared with mammals, insects often produce much less lactate. In locust brain, 10 μmoles/g lactate were measured after 4 hours without oxygen, which is about what an ischemic mouse brain produces in 2 minutes [23].

Figure 5B shows the pathway interpretation of the comparison of simulated hypoxic fluxes. These fluxes are comparisons between adapted and naïve flies *during hypoxia*, and do not represent regulation from normoxia to hypoxia. Pyruvate kinase (PYK), the last step in glycolytic formation of pyruvate, is representative of the lower glycolytic flux in adapted flies. Pyruvate fermentation to alanine by alanine transaminase (ALAT) is active in controls but shut down almost completely in adapted flies, but lactate production from lactate dehydrogenase (LDH), shown for comparison, is similar among the groups. Pyruvate carboxylase (PC), an anaplerotic reaction producing oxalacetate from pyruvate, is less by nearly half in adapted flies, while pyruvate dehydrogenase (PDH) and acetate production from acetyl-CoA synthase (ACS) are greater in the adapted population.

The electron transport chain also shows important differences among the groups. During hypoxia, adapted flies utilize Complex I (NADH dehydrogenase) at a higher rate, while naïve flies rely more on Complex II (succinate dehydrogenase) activity. The Complex II flux in naïve flies is driven by the glycerol phosphate shuttle (GPDH), which transports accumulated cytosolic reducing equivalents in the form of NADH to the mitochondria in the form of FADH_2_. A reducing equivalent entering the electron transport chain via Complex I generates more ATP and consumes an additional proton, compared with one entering via Complex II. Experiments in isolated mitochondria have also demonstrated that activation of Complex II produced a lower P/O ratio (ATP produced per oxygen consumed) than Complex I [20].

## Discussion

Previously, Zhou et. al. used experimental selection over several generations to adapt a *Drosophila *population to be able survive chronic hypoxia. These flies are also able to recover more quickly after acute hypoxia than "naïve" control flies. Adaptation to hypoxia is a remarkable feat for directed evolution over a relatively small number of generations, considering the complexity and scale of cellular mechanisms involved in oxygen regulation.

We studied metabolic aspects of this adaptation, first measuring metabolic concentration profiles using ^1^H NMR spectroscopy. Principle Component Analysis (PCA) of concentration profiles suggested that these genetic adaptations are optimized for a single oxygen concentration, since metabolic profiles of adapted flies raised in normoxia closely resemble the metabolic signatures of acute hypoxic stress, rather than resembling the signatures of either group in their normal steady state (i.e. adapted flies in chronic hypoxia or control flies in normoxia). In other words, moving hypoxia-adapted flies to normoxia causes a "perceived hyperoxia" that induces metabolic stress. As these flies grew under normoxia through their entire life cycle, the stress-like behavior in metabolism may reflect the genetic differences rather than post-translational adaptation, although the gene(s)/mechanism(s) remain to be determined.

Although multivariate analysis has little to say about detailed molecular mechanisms of hypoxia tolerance, the results from PCA extracted a list of metabolites that may play an important role in these tolerance mechanisms. Statistical correlations between specific metabolites and experimental groups cannot be interpreted as causation, since these changes can be indirect effects of upstream regulation. However, the fact that the metabolic profiles showed consistent patterns across gender and experimental conditions suggests that there are general properties to be discovered in metabolic regulation of adapted flies. To investigate differences at the level of pathway activity, we fitted a network model of fly metabolism to fluxes estimated from the time course of metabolite profiles.

### Model-generated hypotheses for improved hypoxia tolerance

When we fitted a flux-balance model to metabolic profiles, simulations of hypoxic metabolism in adapted flies performed better than the control population by specific measures of hypoxia tolerance relating ATP production, oxygen and substrate uptake, and proton production. When the simulations were compared at the network level, the differences in these measures could be traced to increased use of Complex I over Complex II in the mitochondria of adapted flies. Complex I is more efficient by these measures, since P/O ratio and proton uptake are better via this entry point in the electron transport chain [20]. These fluxes co-occur with the uncoupling of glycolysis from mitochondrial pathways during hypoxia in naïve flies, with glycolysis and pyruvate carboxylation to oxalacetate higher and pyruvate dehydrogenase lower as compared to adapted flies.

The summary of pathway differences during simulations of hypoxia are that adapted flies have lower glycolytic flux, greater use of PDH over PC to metabolize glycolytic pyruvate, decreased shuttling of reducing equivalents from glycolysis to the mitochondria, and greater flux through Complex I as compared to the naïve control population. Figure 5B is a cartoon depiction of these pathways.

Additionally, two mechanisms in the model add to the improved ATP generation of adapted flies under acute hypoxia. First, the excess glycolytic activity in naïve flies is diverted through pyruvate carboxylase and into oxalacetate, at a cost of one additional ATP for the conversion plus a pyruvate that would be used more efficiently in another pathway. Second, the use of the acetate pathway results in an additional generation of ATP for each pyruvate consumed.

These conclusions were robust with respect to the choice of oxygen uptake at which we ran the model. Regardless of whether the groups are compared at a common level of ATP production, at a common O_2 _consumption, or whether each group is simulated at its own minimum feasible oxygen uptake, there were clear differences in glycolytic flux and pyruvate carboxylase activity, resulting in heavier use of Complex I and increased ATP generating efficiency in the adapted flies.

In normal conditions, pyruvate carboxylase is an anaplerotic reaction used to replenish oxalacetate, "priming" the TCA cycle after other pathways extract intermediates for biosynthesis. The decreased use of this enzyme during hypoxia in adapted flies might be a result of global suppression of biosynthetic activity.

### Experimental support for the generated hypotheses

The model predictions presented here offer precise mechanistic hypotheses, some of which can be tested experimentally. Specifically, it can be tested whether Complex I activity in the electron transport chain is greater than that of Complex II in adapted flies. In fact, experiments in isolated mitochondria do show downregulation of Complex II in adapted flies [24]. This study also showed decreased oxygen consumption in the mitochondria of adapted flies, which supports the trend of lower minimum oxygen uptake in adapted flies [see Additional file 1, Figure S3].

In simulations, the effects on the electron transport chain were linked to regulation of pyruvate metabolism. The pyruvate branch point was also implicated as the main nexus of hypoxic metabolic regulation in our previous work [12], and in a separate study measuring flux distributions during acute hypoxia in yeast [25]. Low correlation between the global flux response and transcriptional response to acute hypoxia suggested post-transcriptional mechanisms of regulation [25].

**Figure 6:**
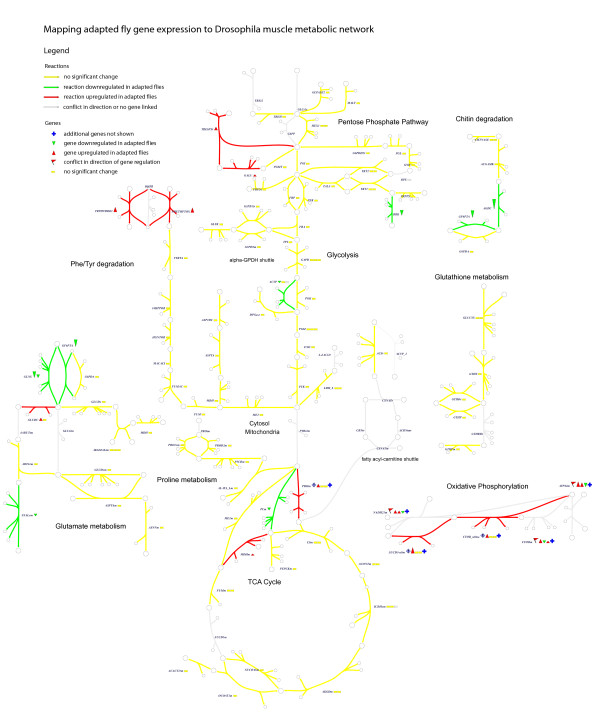
**Gene expression differences between adapted and naïve flies**. Microarray data mapped to the metabolic reconstruction with a threshold of 10% change in expression. At the pyruvate branchpoint, pyruvate carboxylase is downregulated and pyruvate dehydrogenase upregulated in adapted flies, which matches the regulation of fluxes seen in simulation.

Transcriptional data are available for adapted versus naïve flies as well. The microarray data of adapted flies obtained previously [14] showed many changes in gene expression after adaptation to chronic hypoxia, but no individual genes coding for enzymes in our model exceeded their significance threshold. However, connected modules of regulated genes are less likely than individual genes to be significant by chance and thus the same significance threshold is not applicable. Therefore, we mapped the gene expression measurements to our data and explored the effect of a slightly reduced significance threshold (Figure 6). After adjusting the threshold to 10% changes in expression, a cluster of enzymes near the pyruvate branchpoint reaches the threshold. Pyruvate carboxylase is slightly downregulated and pyruvate dehydrogenase is upregulated. In addition, malate dehydrogenase expression is upregulated, which could cause additional inhibition of pyruvate carboxylase by dominating the production of their shared product. Metabolic Control Theory [26] has demonstrated that tighter control of pathway fluxes can be maintained by regulation of demand than supply, i.e. near the end of a pathway. Therefore, this differential control of the fate of pyruvate may be enough to drive the increase in glycolysis, and may also be a target for enhancing hypoxia tolerance.

The effect of hypoxic stress depends on a critical mitochondrial PO_2 _at which, in its simplest form, cellular ATP demand outweighs ATP production by respiratory pathways. Hypoxia-adapted flies are likely to have many changes in oxygen handling and transfer, as well as regulation of ATP-consuming pathways, but together these may have the combined effect of adjusting this critical point in which respiration slows and anaerobic pathways begin to take over. Our analysis of the minimal oxygen required to produce the observed metabolite fluxes provides a hint that this critical PO_2 _may be lower in adapted flies. The fact that physical activity is higher in adapted flies than naïve flies at 4% O_2 _also supports this hypothesis, but ongoing experiments in isolated mitochondria will provide more direct evidence.

## Conclusion

Using a flux-balance model driven by NMR-based metabolomic data, we compared metabolic pathway activity during acute hypoxia between populations of hypoxia-adapted and naïve control flies. Simulations showed higher ATP production, lower proton production, and reduced substrate uptake in hypoxia-adapted flies for equivalent uptake of oxygen.

Our results suggest a systems-level framework for metabolic improvements in adapted flies, which we summarize as follows. Since both populations of flies are in a stupor at 0.5% oxygen, consumption from muscle activity is assumed to be relatively small and we assume that most ATP consumption is from cellular maintenance and biosynthetic processes. Complex I in the electron transport chain produces more ATP and consumes more protons per oxygen and per glucose substrate than Complex II. Also, since Complex I uses mitochondrial NADH and Complex II uses reducing equivalents shuttled from the cytosol, the ratio of I/II is an indicator of the coupling between glycolysis and the TCA cycle. In normoxia, glycolysis and TCA activity are highly coupled, and most pyruvate from glycolysis goes into the TCA cycle. This is the most efficient for the organism in terms of oxygen, ATP, carbohydrate substrate, and protons. These measures of metabolism are important for long-term survival under hypoxia, since [ATP] and pH must be maintained and energy supply should be conserved in any hypoxia tolerant organism. However, biosynthesis and other metabolic demands require carbon intermediates to be taken out of the TCA cycle. To make up for this, glycolysis is increased and pyruvate carboxylase replenishes the carbon at the start of the cycle. The increase in glycolysis is an uncoupling from the TCA cycle that causes greater use of Complex II, and less efficient ATP production (by the above measures).

The model suggests that adapted flies, for each O2 consumed, tend to have decreased glycolysis and pyruvate carboxylase fluxes, and produce acetate rather than oxalacetate. These difference in fluxes are linked to tighter coupling of glycolysis with the TCA cycle, less anaplerotic flux (suggesting less biosynthesis) and more efficient ATP production. Although we present the case of acute hypoxia, it seems probable that similar patterns are present in chronic conditions, since the comparatively small size of adapted flies indicates downregulated anabolic activity [14]. Indeed, suppression of biosynthesis is a common mechanism for surviving hypoxic conditions [8]. The benefits of this overall strategy are reinforced by the recent discovery that hypoxia tolerance in *C. elegans *is improved when protein translation is suppressed [27].

In flux-balance analysis, simulations are parameter free and adhere only to the stoichiometry of the reactions and the law of conservation of mass. Therefore, sources of limitations in these analyses are (1) completeness of the model, e.g. if reactions are improperly compartmentalized, stoichiometrically inaccurate, or omitted from the model altogether; (2) steady state approximation, which assumes that over the time course of the experiment the there is no change in reaction fluxes or metabolite concentrations with the system, and therefore a change in system state happens instantaneously at time zero; (3) the metabolic objective function used to optimize the fluxes; and (4) the quality of the experimental data used to constrain the model.

Our results have significant limitations in all categories. Certain aspects of metabolic control are not able to be modelled, for example the important effects of membrane potential and dynamic control of mitochondrial transport (e.g. proton leak and adenine nucleotide transposase - ANT). However, since the model is compartmentalized, transporters such as ANT are explicitly represented; however, their expression levels are not modeled and therefore fluxes through them are unconstrained. Additionally, concentration levels are not present in the flux model and so concentration-based feedback loops are not considered. As for completeness of model reactions, this was the third iteration of the model and the likelihood that active reactions are included is increasing. Previous time course measurement of metabolic end product concentrations in flies showed a roughly linear increase in during 4 hours hypoxia [12]. The metabolic objective of ATP production is likely to be accurate for such an energy intensive tissue as flight muscle, even though the flies are in a motionless stupor during the experiment. Further, we have focused on differences between two populations, therefore limitations in experimental data and the application of steady state are shared by the control and test populations.

In light of these limitations, this approach is most useful as a platform for discovery, generating a system-level hypothesis for metabolic adaptation to hypoxia, which must be followed up by detailed experiments. The network perspective provides stoichiometrically precise links that connect metabolites, reactions, and cofactors. Therefore, the multiple effects presented are actually a single network-level hypothesis, and a supporting experimental result in one part of the system provides evidence for this overall network activity. In this case, the experimental finding that in adapted flies Complex I activity is greater than Complex II activity during hypoxia [28] supports the network-based hypothesis presented, which is highly interconnected and cannot easily be separated into its components. Gene expression data in Figure 6 provide some additional support. However, it is necessary to perform more detailed experimentation on other components in the system. For example, it would be reasonable to hypothesize that the simulated differences in fluxes of the PDH and PC enzymes between adapted and control populations during acute hypoxia would manifest as differential enzyme activity *in vitro*. Additionally, the ratio of PDH to anaplerotic activity can be adjusted pharmacologically. For example, the PDH kinase inhibitor dichloroacetate has been used to increase PDH flux relative to anaplerosis (via malic enzyme), resulting in improved bioenergetics and contractile function in a mouse model of cardiac hypertrophy [29,30]. This compound could be a candidate for improving *in-vivo *hypoxic recovery in normal flies by slowing anaplerosis through PC, thereby tuning the metabolic network toward the adapted state. Detailed follow-up of these specific hypotheses may lead toward new avenues of investigation.

## Methods

Three separate populations of adapted flies were cultured in hypoxic conditions as described in [14], in parallel with three normoxic control populations. At 3-7 days old, 1-2 groups each of 25 males and 25 females were extracted from each population culture (total of 5 groups male and 5 groups female), subjected to one of the several experimental oxygen conditions described in the text, and snap frozen at the end of the timepoint. For each group of 25 flies, 20 thoraxes were separated from head and abdomen with microforceps on dry ice under a dissecting microscope and stored at -80°C until measurement.

NMR spectra were gathered for each group as follows. Thoraxes were homogenized in an ice bath for 3 minutes in 300 μL of cold 1:1 acetonitrile:water buffer, using an OMNI TH homogenizer. Homogenates were centrifuged in an ice bath (4°C) for 10 minutes at 12,000 RPM. 10 μL of the supernatant was used to determine the total protein concentration by the Bradford methods. For the Bradford assays, samples were diluted 10 times with extraction buffer. The supernatant was ultracentrifuged for 30 minutes at 8,500 RPM using Nanosep centrifugal devices (Pall Life Sciences, Ann Arbor, MI) with a 3 kDa molecular weight cutoff. To reduce the contamination by glycerol, a membrane wetting agent, to below 80 μM, all Nanosep devices were washed 4 times (by 5 minutes centrifugation at 13,000 RPM) with 500 μL deionized water. Filtrate was lyophilized using a vacuum centrifuge for 2 hours at 45°C. Samples were stored at -80°C until measured.

Dried samples were dissolved in 500 μL D_2_O buffered at pH 7.4 with monobasic/dibasic sodium phosphate. The NMR standard TSP (3-trimethylsilyl-^2^H_4_-propionic acid) was added to the samples at a ratio of 1:100 by volume, resulting in a concentration of 0.488 mM. Analyses of samples were carried out by ^1^H NMR spectroscopy on a Bruker Avance 500 operating at 500.13 MHz 1H resonance frequency. The NMR probe used was the 5 mm TXI 1H/2H-13C/15N Z GRD. All NMR spectra were recorded at 25°C. Typically ^1^H were measured with 512 scans into 16384 data points, resulting in an acquisition time of 1.36 seconds. A relaxation delay of 2 seconds additionally ensured T1 relaxation between successive scans. Solvent suppression was achieved via the Noesypresat pulse sequence (Bruker Spectrospin Ltd.) in which the residual water peak is irradiated during the relaxation and mixing time of 80 μs. All ^1^H spectra were manually corrected for phase and baseline distortions within XWINNMR™ (version 2.6, Bruker Spectrospin, Ltd.). Two-dimensional NMR methods including homonuclear correlation spectroscopy (TOCSY) and heteronuclear single quantum correlation spectroscopy (HSQC) were carried out in order to identify and subsequently confirm the assessment of metabolites. Peaks in the 1D spectra were identified, aligned, and quantified by "targeted profiling" algorithms (Weljie et al., 2006) within the software Chenomix NMR Suite 4.5 (Chenomix, Inc.). The list of metabolites discovered in the 2D spectra was used to guide quantification in one dimension.

### Standards

In NMR spectra, absolute concentrations can be obtained from peak integrals if the sample contains an added internal standard of known concentration, or if the concentration of a substance is known by independent means (e.g., glucose determination by biochemical assay) [31]. Scaling factors obtained previously [13] were used to determine absolute concentration of the 10 metabolites included in the model (alanine, lactate, acetate, glutamine, glutamate, glucose, pyruvate, proline, oxaloacetate and 4-aminobutyrate).

### Normalization and scaling

Individual samples within groups were normalized by the sum of all metabolite concentrations in the sample, and then re-scaled by the group average of these concentration totals. Normalization between groups was performed using Bradford assays of the soluble protein content. To minimize the effect of high variability in the Bradford assays, metabolite concentrations for each group of 5 samples were divided by their median protein content. Selected metabolites were scaled empirically using standards (described above) in order to account for small variations in the scaling relationships between peak area in the spectrum and metabolite concentration.

### Principal Component Analysis

For the Principal Component Analysis, all metabolites with at least one measurement above 0.01 mM were included in the dataset. Data from all samples (young and old; control, hypoxia and recovery) were combined into one matrix and principal components were computed using the *princomp *function in Matlab (Mathworks, Inc., Cambridge, MA). Principal component scores for the samples were plotted and visualized within Matlab. The weights of each PC were calculated as the percent each eigenvalue contributes to the sum of all eigenvalues (N = 35).

### Flux-balance analysis

Metabolic fluxes in control and adapted flies were modeled using flux-balance analysis within a genome-wide reconstruction, as described previously [12,13]. Metabolite concentrations for the short term hypoxic conditions (4-hour acute hypoxia) were converted into sets of fluxes by dividing the differences in mean concentrations by the time period, resulting in units of nmol*mg prot^-1^*min^-1^. Standard errors (SE) of the metabolite fluxes were calculated from SE of the concentrations (using the formula SE_C2_-_C1 _= √[SE_C1_^2 ^+ SE_C2_^2^] for subtracting random variables for concentration C_1 _and C_2_) and converted to the same units. A list of 10 "NMR fluxes" was chosen for constraining the flux-balance simulations, based on magnitude of the fluxes and presence of a feasible pathway within the metabolic reconstruction. Virtual "sinks" with unlimited capacity were created for each of these compounds in order to represent metabolite pools, allowing intracellular accumulation and depletion in case substrates and end products did not perfectly balance. The rates of exchange from these "sinks" into/out of the system of reactions were forced to the flux rates calculated from the data. The models with flux constraints are provided [see Additional file 3].

Flux-balance analysis was used to simulate system flux distributions during acute (4-hour) hypoxia for each group. The objective function for the system in all simulations was the reaction representing utilization of ATP via hydrolysis.

### Statistics

Flux constraints from the NMR data were applied to the model with their respective error distributions. Pseudo-random sets of fluxes were created by sampling from normal distributions with mean and standard errors equal to those of each NMR flux applied to the model. A set of 100 different pseudo-random flux constraints were generated and simulated for each experiment. Simulating each of the 100 constraint conditions generated a statistical distribution for each reaction flux output, from which p-values were calculated. Comparisons of individual fluxes across groups were by one-way ANOVA followed by Tukey's multiple comparison test.

### Software

The SimPheny software platform (Genomatica, Inc., San Diego) was used for building the model, visualizing fluxes superimposed on the metabolic network, and mapping the network to microarray data. The model was exported to Matlab (Mathworks, Inc., Cambridge MA) for more detailed flux analysis and statistical testing.

We used Matlab to analyze the sensitivity of flux distributions to variance in the data. The publicly available COBRA toolbox for constraint-based analysis [32], running the freely distributed GNU Linear Programming Kit (GLPK) as a back end solver, was used to import the SimPheny simulations and perform flux-balance analysis within Matlab. M-files for processing NMR data, performing simulations, analyzing flux distributions, and plotting the results have been provided [see Additional file 4].

## List of abbreviations used

NMR: nuclear magnetic resonance; PCA: principal component analysis; PC: principal component; TCA: tricarboxylic acid (cycle); ATP: adenosine triphosphate; O_2_: molecular oxygen; CO_2_: carbon dioxide; PYK: pyruvate kinase; ACS: acetyl-CoA synthase; ALAT: alanine transaminase (cytosolic); LDH: lactate dehydrogenase; PC: pyruvate carboxylase; PDH: pyruvate dehydrogenase; PDHK: pyruvate dehydrogenase kinase; MDH: malate dehydrogenase (mitochondrial); GPDH: glycerol-3-phosphate dehydrogenase shuttle; Complex II: succinyl CoA dehydrogenase (electron transport chain); Complex I: NADH dehydrogenase.

## Authors' contributions

JDF carried out all computational and statistical analysis and drafted the manuscript. LC prepared samples, gathered NMR spectra and calculated metabolite concentrations. DZ cultured the fly populations and carried out the hypoxia stimulus experiments. GGH, GP, and ADM conceived of the study, participated in its design and coordination and helped to draft the manuscript. All authors read and approved the final manuscript.

## Supplementary Material

Additional file 1 **Supplementary Figures**. A text file including Supplementary Figures is included in pdf format. Click here for file

Additional file 2 **Metabolomics data**. All NMR concentration data are provided in the Excel file "NMR_data.xls" Click here for file

Additional file 3 **Models**. The genome-scale model of *Drosophila *central metabolism is provided in SBML format in the file "fly_model.xml" and will be submitted to the Biomodels database at . Click here for file

Additional file 4 **Matlab scripts**. M-files for normalization and principal component analysis of metabolite profiles, and for running and visualizing simulations, are provided in the zipped file "m-files.zip". Model files suitable for Matlab import are included. The COBRA Toolbox version 1.3.3 is required. The top-level script for the analysis is named "adapted_analysis.m." These scripts have not been tested for distribution and are provided without support for informational purposes only. Click here for file
