# Supplementary material for: Metabolism as means for hypoxia adaptation: metabolic profiling and flux balance analysis
Source: BMC Syst Biol. 2009 Sep 9;3:91. doi: 10.1186/1752-0509-3-91 (PMC2749811; doi:10.1186/1752-0509-3-91)
Supplement: Additional file 1 — Supplementary Figures. A text file including Supplementary Figures is included in pdf format. [file 1752-0509-3-91-S1.pdf]

Supplementary Material

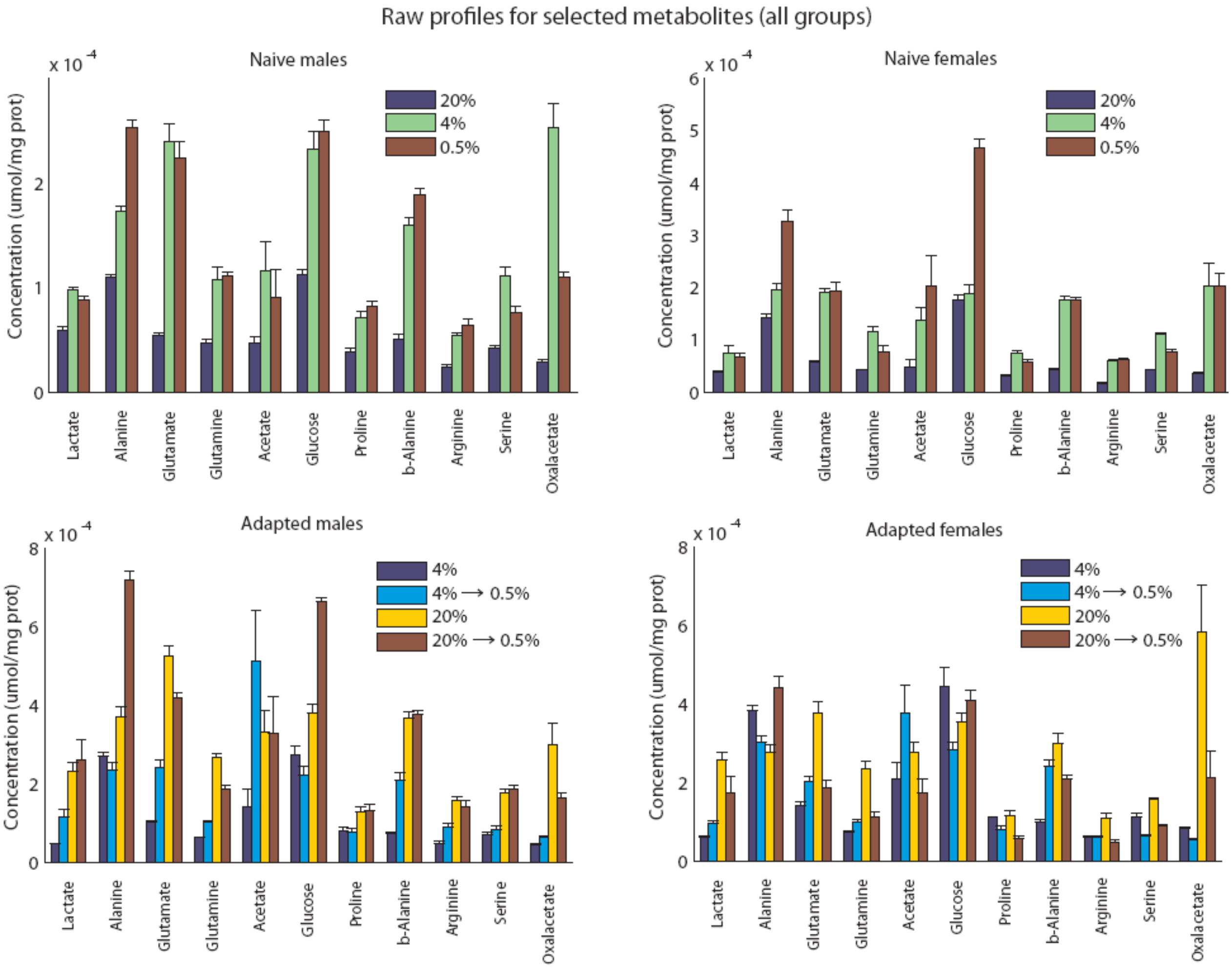

Figure S1: Raw metabolite profiles, for selected metabolites over all experimental groups.

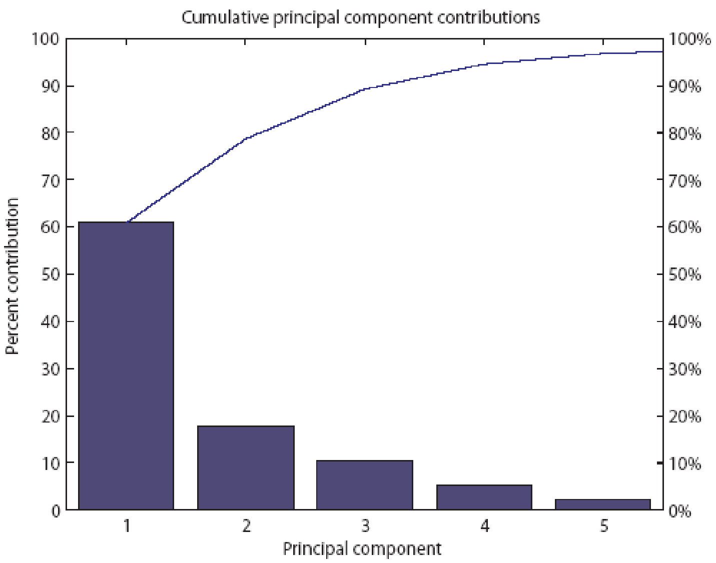

A

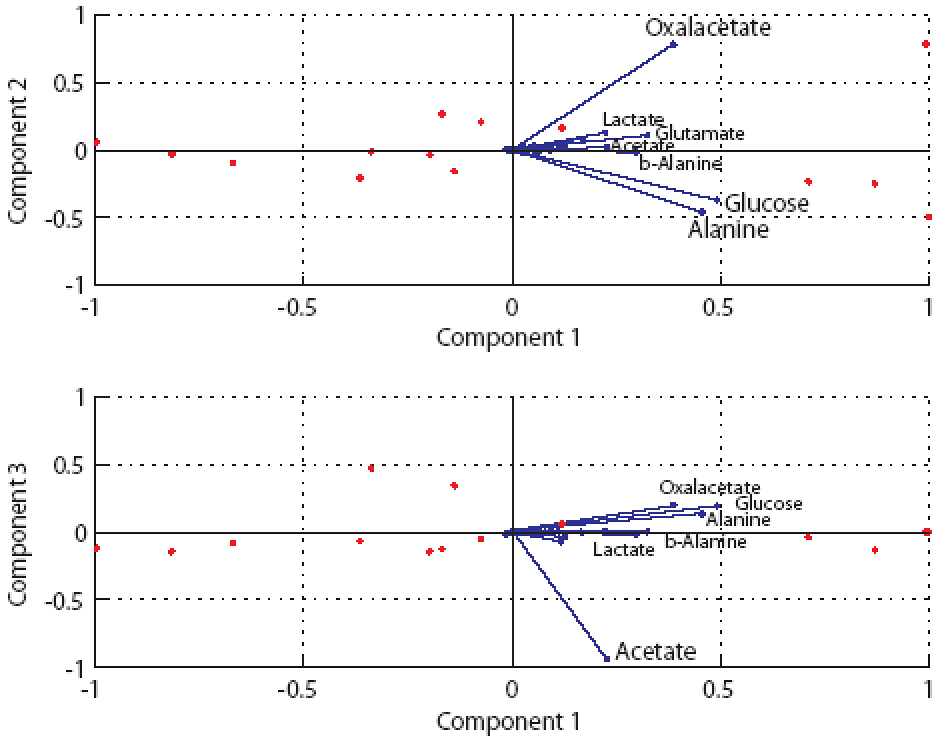

B

Figure S2: Principal component analysis of all metabolite profiles.

A) Contribution of principal components (PCs). PC 1 causes approximately 60% of the variation, and PCs 1 through 3 account for approximately 90% of the differences among groups.

B) Mapping metabolites to PCs. PC 1 represents global increases, PC 2 represents oxalacetate, alanine, and glucose, and PC 3 represents acetate.

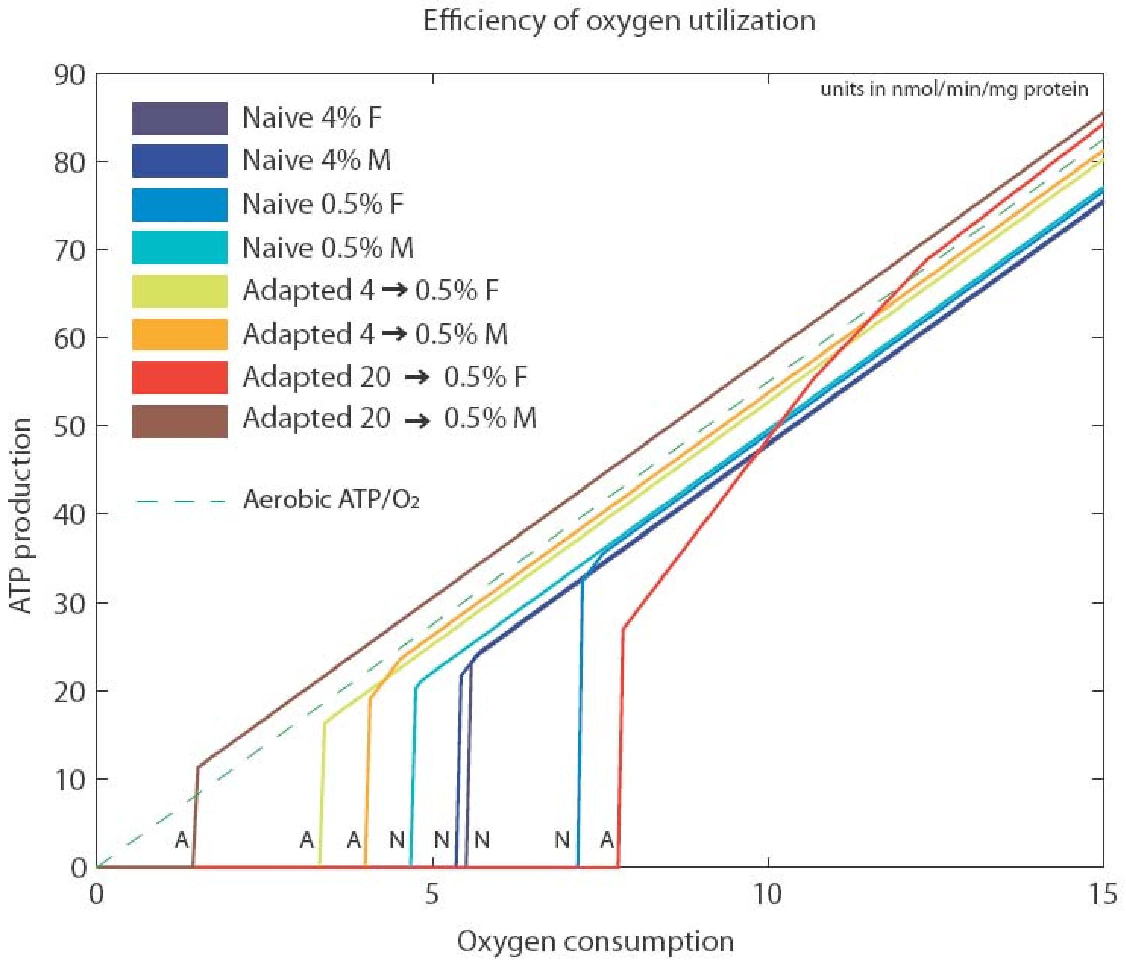

Figure S3: Efficiency of oxygen utilization in terms of ATP production. In adapted flies, ATP-O_2 curves were shifted in the direction of more ATP produced for each unit of oxygen.
